# Quantitative Assessment of Tumor-Infiltrating Lymphocytes Using Machine Learning Predicts Survival in Muscle-Invasive Bladder Cancer

**DOI:** 10.3390/jcm11237081

**Published:** 2022-11-29

**Authors:** Qingyuan Zheng, Rui Yang, Xinmiao Ni, Song Yang, Panpan Jiao, Jiejun Wu, Lin Xiong, Jingsong Wang, Jun Jian, Zhengyu Jiang, Lei Wang, Zhiyuan Chen, Xiuheng Liu

**Affiliations:** 1 Department of Urology, Renmin Hospital of Wuhan University, Wuhan 430060, China; 2 Institute of Urologic Disease, Renmin Hospital of Wuhan University, Wuhan 430060, China; 3 Department of Pathology, Renmin Hospital of Wuhan University, Wuhan 430060, China

**Keywords:** tumor-infiltrating lymphocytes, whole-slide image, machine learning, prognostic marker, muscle-invasive bladder cancer

## Abstract

(1) Purpose: Although assessment of tumor-infiltrating lymphocytes (TILs) has been acknowledged to have important predictive prognostic value in muscle-invasive bladder cancer (MIBC), it is limited by inter- and intra-observer variability, hampering widespread clinical application. We aimed to evaluate the prognostic value of quantitative TILs score based on a machine learning (ML) algorithm to identify MIBC patients who might benefit from immunotherapy or the de-escalation of therapy. (2) Methods: We constructed an artificial neural network classifier for tumor cells, lymphocytes, stromal cells, and “ignore” cells from hematoxylin-and-eosin-stained slide images using the QuPath open source software. We defined four unique TILs variables based on ML to analyze TILs measurements. Pathological slide images from 133 MIBC patients were retrospectively collected as the discovery set to determine the optimal association of ML-read TILs variables with patient survival outcomes. For validation, we evaluated an independent external validation set consisting of 247 MIBC patients. (3) Results: We found that all four TILs variables had significant prognostic associations with survival outcomes in MIBC patients (*p* < 0.001 for all comparisons), with higher TILs score being associated with better prognosis. Univariate and multivariate Cox regression analyses demonstrated that electronic TILs (eTILs) variables were independently associated with overall survival after adjustment for clinicopathological factors including age, sex, and pathological stage (*p* < 0.001 for all analyses). Results analyzed in different subgroups showed that the eTILs variable was a strong prognostic factor that was not redundant with pre-existing clinicopathological features (*p* < 0.05 for all analyses). (4) Conclusion: ML-driven cell classifier-defined TILs variables were robust and independent prognostic factors in two independent cohorts of MIBC patients. eTILs have the potential to identify a subset of high-risk stage II or stage III-IV MIBC patients who might benefit from adjuvant immunotherapy.

## 1. Introduction

Bladder cancer (BCa) is the most common malignancy of the urinary system and one of the top ten cancers worldwide, with approximately 573,000 new cases and 213,000 deaths worldwide in 2020 [1]. As the tumor invades different layers of the bladder, the overall survival (OS) of patients with muscle-invasive bladder cancer (MIBC) significantly declines, with the 5-year relative survival rate falling to 14% for those with stage IV bladder cancer [2,3]. Although neoadjuvant chemotherapy and immunotherapy can reduce the risk of distant spread in MIBC patients, 5–10% of patients remain unresponsive, resulting in potentially fatal surgical delays and treatment toxicity [4,5]. Accordingly, it is crucial to develop prognostic factors that might benefit high-risk patients who can benefit from adjuvant therapy and low-risk patients who can be safely spared further treatment. Tumor–node–metastasis (TNM) staging, established by the American Joint Committee on Cancer (AJCC), as well as pathological grading, and biomolecular markers such as FGFR and Ki67 are traditional prognostic markers for BCa patients. However, the development of new markers has been slow over the last two decades [6]. The tumor microenvironment (TME) has received increasing attention for its role in the development and progression of solid tumors, and is now involved in the development of new and meaningful prognostic markers [7]. Tumor-infiltrating lymphocytes (TILs) and fibroblasts present in the tumor stroma have been shown to be involved in a wide range of signaling interactions in the TME with cancer cells and can serve as targets for optimizing subsequent treatment decisions [8,9].

TILs have high prognostic value in many solid tumors. Overall, a high abundance of TILs tends to be associated with better prognosis [10]. Several studies in BCa have supported this hypothesis. Liu et al. [11] revealed that elevated TILs were associated with longer survival in BCa patients and were an important marker for predicting prognosis. Shi et al. [12] demonstrated that a high CD3D/CD4 (total T cells/helper T cells) ratio could better predict survival in MIBC patients. In 2014, the International TILs Working Group (ITWG) published a standardized method for evaluating TILs score in breast cancer pathological slide images [13]. Since then, TILs of most solid tumors have mainly relied on semi-quantitative assessment by pathologists according to this standard with the naked eye or microscope, typically using the predefined cut-point of TILs as the evaluation standard [14,15]. Ledderose et al. [16] revealed that increased stromal lymphocyte infiltration was related to significantly higher OS, tumor-specific survival, and disease-free survival in MIBC patients when using the ITWG-recommended approach. However, this traditional visual assessment method is susceptible to inter-observer or inter-institutional variability resulting in a lack of reproducibility and requiring review by experienced pathologists. This means that the TILs scoring technique cannot be widely used in clinical practice [17]. Hence, there is an urgent need to develop an automated assistance system for quantitative, reliable and reproducible analysis of pathological slide images.

In recent years, the development of digital pathology imaging and advances in machine learning (ML) have greatly facilitated the discovery of cancer biomarkers. A growing number of computer image analysis methods are being developed for commercial or free open source use, such as QuPath [18]. ML computational methods, based on pathological images, have been widely used in a variety of tumors, including breast cancer [19], lung cancer [20], melanoma [21], and colorectal cancer [22]. ML allows the extraction of higher-order features in pathological images in a hand-predefined manner to explore quantitative assessments of the interaction between tumors and the TME. However, a ML-based quantitative calculation method of TILs in BCa has not been reported, and it is worth further exploring the prognostic value of the quantitative results of TILs in MIBC patients.

In this study, we developed a pipeline for automatic analysis of TILs in pathological images based on ML technology and QuPath open source software for prognostic prediction of MIBC patients. The goal of this work is not only to help pathologists more accurately assess TILs, but also to validate the potential pathologist-independent utility of this prognostic analysis method in a clinical setting to determine which MIBC patients might benefit from immunotherapy. Finally, we demonstrated in two cohorts that TILs are a powerful independent prognostic factor that can assist clinicians in risk stratification of MIBC patients and facilitate personalized treatment.

## 2. Materials and Methods

### 2.1. Clinical Cohorts

We retrospectively evaluated independent cases from two cohorts from the following publication and institution, respectively: (a) The Cancer Genome Atlas (TCGA) and (b) Renmin Hospital of Wuhan University (RHWU; Wuhan, Hubei, China). Pathological images for both cohorts were rendered in whole-slide image (WSI) format. The TCGA cohort included 457 WSIs of 386 BCa patients, and each patient may have multiple WSIs (https://portal.gdc.cancer.gov/repository, accessed on 24 November 2022). The RHWU cohort included 150 WSIs of 150 MIBC patients.

**Figure 1:**
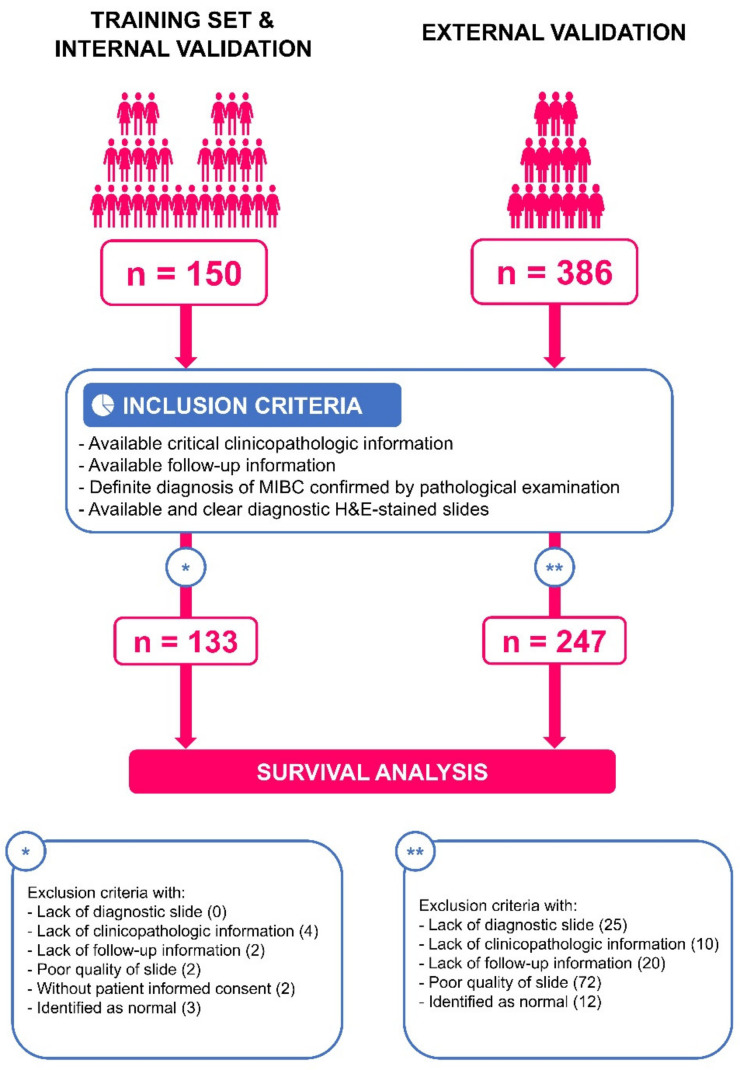
Inclusion and exclusion criteria for both cohorts. The algorithm was first developed and internally validated in MIBC patients from RHWU. External validation was then performed with patients from TCGA.

Inclusion criteria for both cohorts were as follows: (a) available clinicopathological information; (b) available follow-up information; (c) definitive pathological diagnosis of MIBC; (d) diagnostic slides, not tissue slides; (e) availability of clear Hematoxylin-and-Eosin (H&E)-stained pathological slides (Figure 1).

Clinical information, biological and pathological characteristics of patients in the TCGA cohort were collected through the UCSC Xena database (https://xenabrowser.net/datapages/, accessed on 24 November 2022), and those in the RHWU cohort were obtained through the hospital management system and follow-up. Patients in both cohorts were reclassified according to the eighth edition of the TNM staging system developed by the AJCC [23].

### 2.2. Ethics

This retrospective study was approved by the RHWU Ethics Committee (No. WDRY2022-K084) and the informed consent of the patients was obtained.

### 2.3. Digital Scanning

H&E-stained slides of the RHWU cohort were digitally scanned using a KF-PRO-020 digital scanner at ×20 magnification (0.5 µm per pixel). After scanning, urological pathologists performed a careful review of all WSIs, ensuring that all images were clear and not blurred before image analysis, and then annotated the tumor areas. Since the WSIs of the two cohorts have different magnifications—specifically, the original magnification of the WSIs of the TCGA cohort was 40× (no fixed size, up to 100,000 × 100,000 pixels), while the original magnification of the RHWU was 20×—we uniformly processed these images to 20× magnification and used them to develop the next algorithm.

### 2.4. WSI Image Analysis

**Figure 2:**
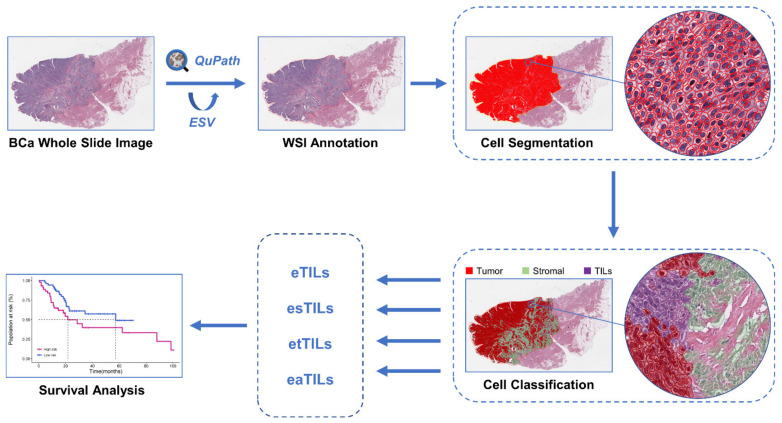
The flow chart of histopathology image processing and analysis based on machine learning in this study. BCa, bladder cancer; WSI, whole-slide image. ESV, estimated staining vector; TIL, tumor-infiltrating lymphocyte.

Image analysis for all WSIs was performed using QuPath v0.3.2 (https://qupath.github.io/, accessed on 24 November 2022), an open source digital image analysis software platform with built-in trainable ML algorithms. All WSIs were quality-checked before being imported into QuPath. First, due to differences in slide staining between and within institutions, the “estimated staining vector” function in QuPath was used to optimize H&E staining estimates for each WSI. We used watershed cell detection [24] to identify and segment all cells within the tumor area with the following parameters: Detection image: hematoxylin OD; requested pixel size: 0.5 µm; background radius: 8 µm; median filter radius: 0 µm; sigma: 1.5 µm; minimum cell area: 10 µm^2^; maximum cell area: 400 µm^2^; threshold: 0.1; maximum background intensity: 2. The quality of cell segmentation was controlled by urological pathologists. Representative specific regions were then selected to classify tumor cells (red), immune cells (purple), and stroma cells (green), and the remaining irrelevant factors (false detection and background) were set to “ignore”. We applied a built-in neural network [25] classifier with eight hidden layers (maximum iterations: 1000) to train to produce the best cell classification, adding additional specific regions as needed during training to improve classification accuracy. Furthermore, to further increase the classification accuracy, we also added smoothed object features at 25 µm and 50 µm radius to complement the existing measurement features of cells. Features used in cell classification are described in Supplementary Table S1. The training of the classifier required multiple rounds of optimization to achieve the best classification effect, which was quality-controlled by urological pathologists. Then, with the help of automated scripts, the trained classifier was applied to all WSIs, and the number of cells and area of each type were counted in preparation for the quantitative evaluation of TILs. Finally, we established a flow chart for the quantitative analysis of TILs in pathological images based on the QuPath open source software (Figure 2).

### 2.5. Assessment of TILs Using Four Variables

In this study, ML-defined TILs were constructed using four different methods, namely eTILs%, esTIL%, etTILs% and eaTILs (mm^2^). The specific definitions were as follows:

(1) Calculate the proportion of TILs in tumor cells: eTILs% = (TILs/TILs + tumor cells) × 100
(2) Calculate the proportion of TILs in stromal cells: esTILs% = (TILs/TILs + stromal cells) × 100
(3) Calculate the proportion of TILs in total cells: etTILs% = (TILs/total cells) × 100
(4) Calculate the infiltration density of TILs in the tumor region: eaTILs = TILs/tumor region areas analyzed (mm^2^)

### 2.6. Statistical Analyses

For statistical analysis, SPSS 26.0 software (SPSS Inc., Chicago, IL, USA) was used. The statistically significant thresholds for the four variables of TILs were determined using X-tile cut-point finder [26], a software that traverses possible combinatorial partitions to find the best classification threshold. For prognostic analysis, Kaplan–Meier survival curves were drawn using R software (Rx64 3.5.1) and a log-rank test was performed. The prognostic value of TILs variables was assessed using univariate and multivariate Cox proportional hazards models. For Cox multivariate analysis, a Cox model was generated using eTILs, age, sex, lymphovascular invasion and TNM stage as predictors. All statistical tests were two-tailed and significance was expressed as (*) *p* < 0.05, (**) *p* < 0.01, (***) *p* < 0.001, (****) *p* < 0.0001.

## 3. Results

### 3.1. Patient Characteristics

After screening for inclusion, we included 247 MIBC patients in the TCGA cohort and 133 MIBC patients in the RHWU cohort, and only one representative WSI was selected for each patient for analysis. The pathological types of patients in both cohorts were transitional cell carcinomas. Table 1 presents the baseline characteristics of the two cohorts. We used the RHWU cohort as the training set and TCGA as the external validation set to verify the robustness of the algorithm.

### 3.2. Measurement of eTILs as a Prognostic Variable in Two Cohorts

**Figure 3:**
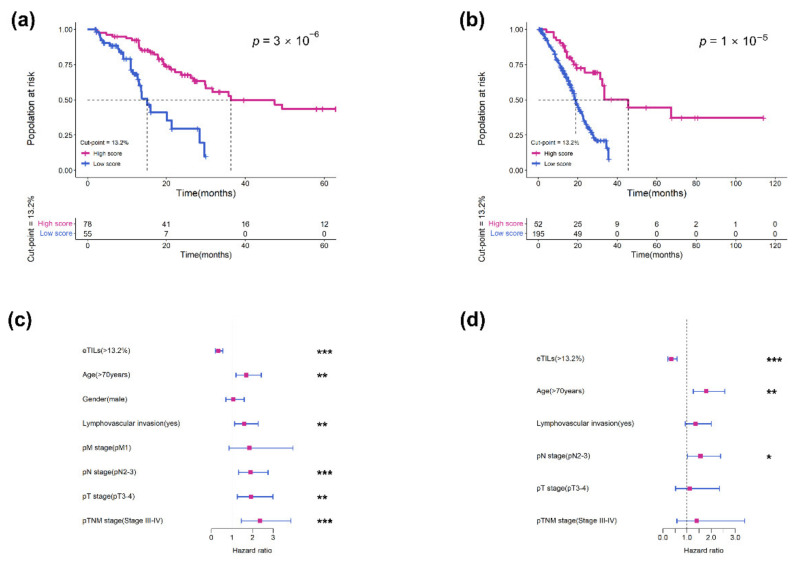
Measurement of eTILs with a 13.2% cut-point as a prognostic variable in two cohorts. Kaplan–Meier survival curves for (**a**) RHWU and (**b**) TCGA cohort. Hazard ratio and 95% confidence interval for eTILs and other clinicopathological features to predict survival in (**c**) univariate Cox and (**d**) multivariate Cox analyses. ***, *p* < 0.001; **, *p* < 0.01; *, *p* < 0.05.

Measurement of eTILs using an ML-based cell classifier and a cut-point of 13.2% identified in both cohorts indicated that a high level of eTILs was associated with longer OS in the RHWU cohort (hazard ratio [HR] = 0.264, *p* < 0.0001; Figure 3a) and TCGA cohort (HR = 0.333, *p* < 0.0001; Figure 3b). We then performed univariate and multivariate Cox analyses in the TCGA cohort to assess the association of eTILs and clinicopathological features with prognosis (Table 2). In univariate Cox analysis, eTILs (with a predefined 13.2% cut-point), age, lymphovascular invasion, pT stage, pN stage, and pTNM stage were all significantly associated with OS (Figure 3c). Multivariate Cox analysis showed that eTILs remained a significant prognostic factor after retaining the important prognostic indicators in univariate analysis (HR = 0.345, log-rank *p* < 0.001; Figure 3d and Table 2).

### 3.3. Validation of the Prognostic Effect of eTILs in Different Subgroups

**Figure 4:**
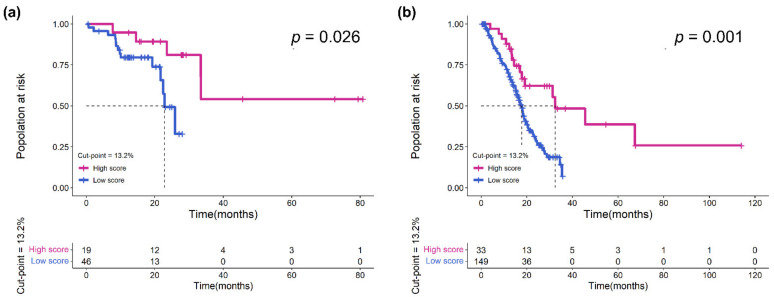
Assessment of eTILs in pTNM stage II and pTNM stage III-IV of TCGA cohort. (**a**) Kaplan–Meier curve of overall survival in stage II and (**b**) Kaplan–Meier curve of overall survival in stage III–IV by eTILs dichotomized at 13.2%.

**Figure 5:**
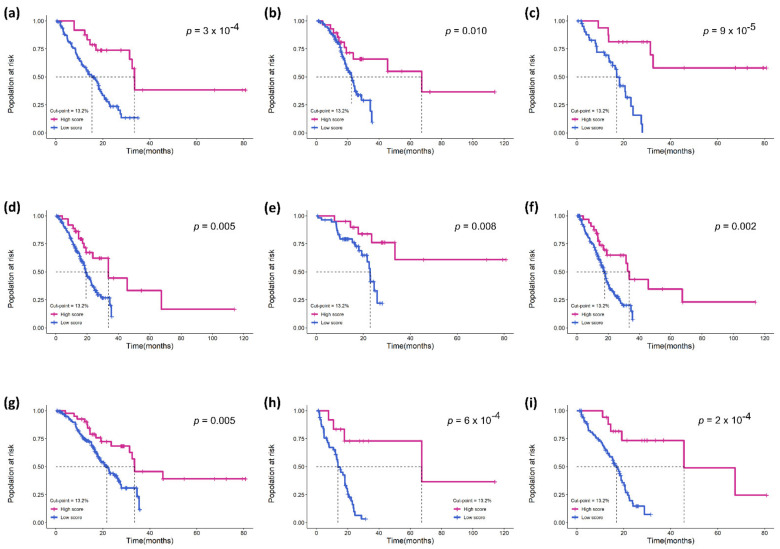
The performance of eTILs with a 13.2% cut-point in predicting prognosis in the TCGA cohort. Kaplan–Meier survival curves for the following subgroups: (**a**) age ≥ 70; (**b**) age < 70; (**c**) male; (**d**) female; (**e**) pT stage 2; (**f**) pT stage 3–4; (**g**) pN stage 0–1; (**h**) pN stage 2–3; (**i**) lymphovascular invasion.

To verify that eTILs could be used for risk identification of MIBC patients of different stages, we used eTILs with a 13.2% cut-point to analyze patients in pTNM stage II and pTNM stage III–IV in the TCGA cohort, and the results confirmed that higher eTILs were not only associated with better prognosis in Stage III and IV (*p* = 0.001; Figure 4b) but also had a prognostic value in stage II (*p* = 0.026; Figure 4a). Further, we also predicted survival after stratification with other characteristics (e.g., age, sex, pT stage, pN stage, pTNM stage, histological grade, and lymphovascular invasion; Figure 5). These results demonstrated that eTILs were a powerful prognostic factor that was not redundant with pre-existing clinicopathological features and was an effective prognostic method independent of current AJCC TNM staging.

### 3.4. Assessment of Four TIL Variables in TCGA Cohort

**Figure 6:**
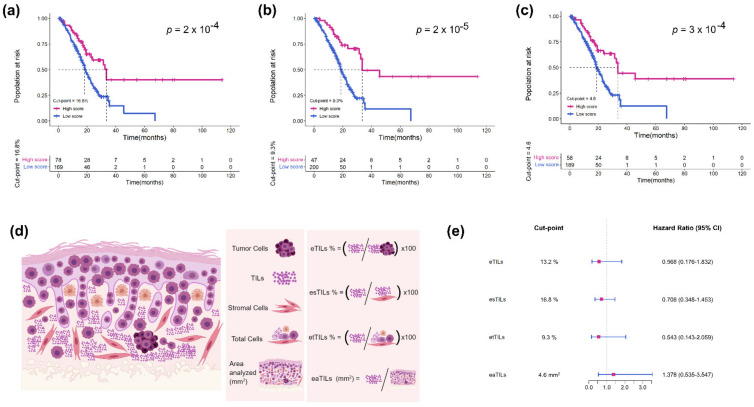
Assessment of four TILs variables including eTILs, etTILs, esTILs, and eaTILs. Kaplan–Meier survival curves for (**a**) etTILs, (**b**) esTILs, and (**c**) eaTILs with optimal cut-points in TCGA cohort. (**d**) Schematic diagram illustrating the four TILs variables. (**e**) Forest plot of the four TILs variables with optimal cut-points in TCGA cohort.

To determine the best approach for specific forms of TILs for potential future clinical adoption, we tested four different approaches to assess the density and proportion of eTILs by cell types and the area analyzed. These TILs variables are shown schematically in Figure 6d. We used the RHWU cohort as a training set to find each possible optimal cut-point and the association of each TILs variable with patient survival outcomes. It was not statistically reasonable to compare the *p* values for the four TILs variables since the goal of this assessment was to examine their performance; nevertheless, HR comparisons might be made to ascertain the relative prognostic strength of each variable. Our results showed that all TILs variables were significantly associated with OS (*p* < 0.001; Figure 6a–c). HRs were similar among eTILs, esTILs and etTILs variables, but eTILs (HR = 0.568, 95% confidence interval [CI] = 0.176–1.832) and etTIL (HR = 0.543, 95% CI = 0.143–2.059) appeared to be more robust methods. This suggested that eTILs and etTILs might perform better than the other two methods in future large prospective or retrospective cohort studies.

## 4. Discussion

Chemotherapy is the first-line treatment for advanced and metastatic BCa, but its objective response rate is unsatisfactory. Immunotherapy is currently available with immune checkpoint inhibitors (ICIs) based on programmed cell death 1 receptor (PD-1) or PD-ligand 1 [27]. Nonetheless, MIBC patients receiving immunotherapy still experience treatment-related adverse events and a severely compromised quality of life [28,29]. It is critical to evaluate the role of immunotherapy as adjunctive therapy for MIBC patients, as this might spare them from immunotherapy toxicity. Our study confirmed in two cohorts of MIBC patients that the eTILs score was an independent prognostic marker in MIBC patients and proved that this effect was present in both low- and high-stage patients receiving adjuvant therapy.

Although current studies have found that TILs may provide prognostic information for MIBC patients [30,31], it has not been widely used in the clinical practice management of MIBC due to inter-institutional and inter-operator variability. Much research has recommended the use of ML or convolutional neural network for the analysis of H&E or immunohistochemistry-based stained slides in breast cancer, melanoma, and lung cancer to solve the problem of poor repeatability due to subjective variations [32,33,34]. Some of these ML methods are based on patch classification, others rely on cell detection and classification. However, none of these ML- or deep-learning-derived quantification methods for TILs has yet been validated in MIBC patients, and they lack clinical experience, which is essential for clinical applicability. Furthermore, different types of TILs variables need to be compared in order to determine the optimum variables for large cohort studies. Here, we demonstrated that ML-calculated TILs score can provide comprehensive information on TILs in TEM of MIBC and differentiate patients with different risks during the same staging phase.

In our study, we developed a cell classifier based on the QuPath platform for quantitative measurement of TILs and derived four different TILs variables [eTILs%, esTIL%, etTILs% and eaTILs (mm^2^)] to represent the proportion and density of TILs in different cell types and areas analyzed. The cell segmentation method we used was unsupervised, and we followed this with artificial neural network-based cell classification. The advantage of this strategy is that it only requires less data to achieve better training results. However, its limitation is that the accuracy of classification and segmentation is susceptible to differences between WSIs, which might result in overfitting of the classifier during training. To address this limitation, we optimized and unified H&E staining for all WSIs, and strictly controlled the quality of segmentation and classification by urologic pathologists to avoid catastrophic phenomena. Finally, we confirmed the prognostic effect of all four TILs variables in both cohorts.

Although our research is still in its early stages, objective evaluation of TILs might have a clinical prognostic effect, allowing clinicians to identify a subset of patients who might benefit from immunotherapy, or otherwise prevent overtreatment. We validated the optimal predefined cut-points for TILs variables with two cohorts, but these cut-points are likely not used in large cohorts because they are not adjusted for specific stage, tumor subtype, and treatment. Accordingly, we propose treating the future TILs score as a continuous variable and adjusting for the aforementioned elements as needed to develop appropriate cut-points for certain situations.

This work has many potential limitations. One of the limitations is that segmentation or classification errors in the algorithm can result in biased variables in the computed TILs. Since tumor cells are highly heterogeneous, they tend to disguise themselves as other cells, making classification more challenging. It is worth noting that etTILs is calculated with all detected cells as the denominator, therefore using etTILs might lessen the inaccuracy caused by this misclassification. In addition, the variant histology of BCa needs further consideration, for example, the small cell component might have some microscopic features resembling TILs. Another limitation is that we did not finetune TILs to differentiate between stromal TILs (sTILs) and intratumoral TILs (iTILs). Although iTILs account for only 1% to 3% of TILs in most circumstances, they are frequently disastrous when they occur. Hence, it is essential for pathologists to rigorously review these WSIs for exclusion or reanalysis in studies. Finally, our most important limitations are that both cohorts are retrospective, that patients in cohorts can have different treatments and that WSIs staining differs between cohorts. Even though pathological images from clinics are restricted, we only employed WSIs for analysis in our work and did not include tissue microarrays (TMA) images like [35]. This is because TMA imaging might contain significantly less information than data extracted from WSI methods, which cannot be fully representative of the prognosis of the whole tissue image. However, further research is required to demonstrate the difference between the two.

## 5. Conclusions

In conclusion, we validated that the eTILs score was a strong independent prognostic marker in MIBC patients and was significantly associated with OS. The developed ML algorithm has the potential to be a convenient and useful quantitative tool for risk stratification. Through subgroup analysis, we confirmed that the eTILs score can assist clinicians in identifying MIBC patients who might benefit from immunotherapy or avoid overtreatment. In the future, TILs variables will be complemented with molecular typing of cells, and larger prospective cohort studies will be conducted to find the best approach for the subset of MIBC patients who might benefit from immunotherapy.

## Figures and Tables

**Table 1:** Clinical, biological, and pathological features of the MIBC patients included in this study.

	RHWU (N = 133)	TCGA (N = 247)
Age (years)	66 (26, 87)	69 (37, 90)
Sex		
female	20 (15.04%)	60 (24.29%)
male	113 (84.96%)	187 (75.71%)
pT stage		
pT2	52 (39.10%)	70 (28.34%)
pT3	63 (47.37%)	131 (53.04%)
pT4	18 (13.53%)	40 (16.19%)
pTx	0 (0%)	6 (2.43%)
pN stage		
pN0	66 (49.62%)	141 (57.09%)
pN1	34 (25.56%)	29 (11.74%)
pN2	18 (13.53%)	58 (23.48%)
pN3	15 (11.28%)	5 (2.02%)
pNx	0 (0%)	14 (5.67%)
pM stage		
pM0	129 (96.99%)	100 (40.49%)
pM1	4 (3.01%)	7 (2.83%)
pMx	0 (0%)	140 (56.68%)
pTNM stage		
Stage II	38 (28.57%)	65 (16.31%)
Stage III	74 (55.64%)	86 (34.82%)
Stage IV	21 (15.79%)	96 (38.87%)
Lymphovascular invasion		
No	83 (62.41%)	90 (33.21%)
Yes	50 (37.59%)	99 (36.53%)
Missing	0 (0%)	82 (30.26%)
Survival status		
Alive	77 (57.89%)	121 (48.99%)
Dead	56 (42.11%)	126 (51.01%)
OS time (months)	15.3 (1.9, 66.0)	17.5 (0.5, 114.0)

MIBC, Muscle-invasive Bladder Cancer; RHWU, Renmin Hospital of Wuhan University; TCGA, The Cancer Genome Atlas.

**Table 2:** Cox analyses of prognostic factors in the TCGA cohort.

	Univariate Analysis		Multivariate Analysis	
	HR (95%CI)	*p* Value	HR (95%CI)	*p* Value
Age				
<70	Ref.		Ref.	
≥70	1.702 (1.195, 2.424)	<0.001	1.798 (1.259, 2.567)	0.001
Gender				
female	Ref.			
male	1.058 (0.703, 1.591)	0.788		
pT stage				
pT1–2	Ref.		Ref.	
pT3–4	1.938 (1.258, 2.985)	0.003	1.111 (0.526, 2.345)	0.783
pN stage				
pN0–1	Ref.		Ref.	
pN2–3	1.907 (1.323, 2.749)	<0.001	1.557 (1.014, 2.391)	0.043
pM stage				
pM0	Ref.			
pM1	1.849 (0.859, 3.981)	0.116		
pTNM stage				
Stage II	Ref.		Ref.	
Stage III–IV	2.438 (1.591, 3.876)	<0.001	1.407 (0.581, 3.403)	0.449
Lymphovascular invasion				
No	Ref.		Ref.	
Yes	1.603 (1.128, 2.277)	0.008	1.360 (0.924, 2.003)	0.119
eTILs				
Low eTILs	Ref.		Ref.	
High eTILs	0.333 (0.199, 0.558)	<0.001	0.345 (0.203, 0.586)	<0.001

95% CI, 95% Confidence Interval; HR, Hazard Ratio.

## Data Availability

The datasets of TCGA cohort for this study can be found in the [The Cancer Genome Atlas Program] [https://portal.gdc.cancer.gov/, accessed on 24 November 2022].

## Supplementary Materials

The following supporting information can be downloaded at: https://www.mdpi.com/article/10.3390/jcm11237081/s1, Figure S1: (a) In each whole slide image, the entire tumor region of one whole image selected by the pathologists for each patient was annotated and analyzed. The details of tumor region areas analysed in the (b) RHWU cohort and (c) TCGA cohort are shown; Table S1: Features incorporated by neural network-driven cell classifiers.
