# Supplementary material for: Quantitative Assessment of Tumor-Infiltrating Lymphocytes Using Machine Learning Predicts Survival in Muscle-Invasive Bladder Cancer
Source: J Clin Med. 2022 Nov 29;11(23):7081. doi: 10.3390/jcm11237081 (PMC9739988; doi:10.3390/jcm11237081)

Supplementary Materials

Supplementary Table S1

Features incorporated by neural network-driven cell classifiers.

Nucleus: Area
Nucleus: Perimeter
Nucleus: Circularity
Nucleus: Max caliper
Nucleus: Min caliper
Nucleus: Eccentricity
Nucleus: Hematoxylin OD mean
Nucleus: Hematoxylin OD sum
Nucleus: Hematoxylin OD std dev
Nucleus: Hematoxylin OD max
Nucleus: Hematoxylin OD min
Nucleus: Hematoxylin OD range
Nucleus: Eosin OD mean
Nucleus: Eosin OD sum
Nucleus: Eosin OD std dev
Nucleus: Eosin OD max
Nucleus: Eosin OD min
Nucleus: Eosin OD range
Cell: Area
Cell: Perimeter
Cell: Circularity
Cell: Max caliper
Cell: Min caliper
Cell: Eccentricity
Cell: Hematoxylin OD mean
Cell: Hematoxylin OD std dev
Cell: Hematoxylin OD max
Cell: Hematoxylin OD min
Cell: Eosin OD mean
Cell: Eosin OD std dev
Cell: Eosin OD max
Cell: Eosin OD min
Cytoplasm: Hematoxylin OD mean
Cytoplasm: Hematoxylin OD std dev
Cytoplasm: Hematoxylin OD max
Cytoplasm: Hematoxylin OD min
Cytoplasm: Eosin OD mean
Cytoplasm: Eosin OD std dev

Cytoplasm: Eosin OD max
Cytoplasm: Eosin OD min
Nucleus/Cell area ratio
Smoothed: 25 µm: Nucleus: Area
Smoothed: 25 µm: Nucleus: Perimeter
Smoothed: 25 µm: Nucleus: Circularity
Smoothed: 25 µm: Nucleus: Max caliper
Smoothed: 25 µm: Nucleus: Min caliper
Smoothed: 25 µm: Nucleus: Eccentricity
Smoothed: 25 µm: Nucleus: Hematoxylin OD mean
Smoothed: 25 µm: Nucleus: Hematoxylin OD sum
Smoothed: 25 µm: Nucleus: Hematoxylin OD std dev
Smoothed: 25 µm: Nucleus: Hematoxylin OD max
Smoothed: 25 µm: Nucleus: Hematoxylin OD min
Smoothed: 25 µm: Nucleus: Hematoxylin OD range
Smoothed: 25 µm: Nucleus: Eosin OD mean
Smoothed: 25 µm: Nucleus: Eosin OD sum
Smoothed: 25 µm: Nucleus: Eosin OD std dev
Smoothed: 25 µm: Nucleus: Eosin OD max
Smoothed: 25 µm: Nucleus: Eosin OD min
Smoothed: 25 µm: Nucleus: Eosin OD range
Smoothed: 25 µm: Cell: Area
Smoothed: 25 µm: Cell: Perimeter
Smoothed: 25 µm: Cell: Circularity
Smoothed: 25 µm: Cell: Max caliper
Smoothed: 25 µm: Cell: Min caliper
Smoothed: 25 µm: Cell: Eccentricity
Smoothed: 25 µm: Cell: Hematoxylin OD mean
Smoothed: 25 µm: Cell: Hematoxylin OD std dev
Smoothed: 25 µm: Cell: Hematoxylin OD max
Smoothed: 25 µm: Cell: Hematoxylin OD min
Smoothed: 25 µm: Cell: Eosin OD mean
Smoothed: 25 µm: Cell: Eosin OD std dev
Smoothed: 25 µm: Cell: Eosin OD max
Smoothed: 25 µm: Cell: Eosin OD min
Smoothed: 25 µm: Cytoplasm: Hematoxylin OD mean
Smoothed: 25 µm: Cytoplasm: Hematoxylin OD std dev
Smoothed: 25 µm: Cytoplasm: Hematoxylin OD max
Smoothed: 25 µm: Cytoplasm: Hematoxylin OD min
Smoothed: 25 µm: Cytoplasm: Eosin OD mean
Smoothed: 25 µm: Cytoplasm: Eosin OD std dev
Smoothed: 25 µm: Cytoplasm: Eosin OD max
Smoothed: 25 µm: Cytoplasm: Eosin OD min
Smoothed: 25 µm: Nucleus/Cell area ratio

Smoothed: 25 µm: Nearby detection counts
Smoothed: 50 µm: Nucleus: Area
Smoothed: 50 µm: Nucleus: Perimeter
Smoothed: 50 µm: Nucleus: Circularity
Smoothed: 50 µm: Nucleus: Max caliper
Smoothed: 50 µm: Nucleus: Min caliper
Smoothed: 50 µm: Nucleus: Eccentricity
Smoothed: 50 µm: Nucleus: Hematoxylin OD mean
Smoothed: 50 µm: Nucleus: Hematoxylin OD sum
Smoothed: 50 µm: Nucleus: Hematoxylin OD std dev
Smoothed: 50 µm: Nucleus: Hematoxylin OD max
Smoothed: 50 µm: Nucleus: Hematoxylin OD min
Smoothed: 50 µm: Nucleus: Hematoxylin OD range
Smoothed: 50 µm: Nucleus: Eosin OD mean
Smoothed: 50 µm: Nucleus: Eosin OD sum
Smoothed: 50 µm: Nucleus: Eosin OD std dev
Smoothed: 50 µm: Nucleus: Eosin OD max
Smoothed: 50 µm: Nucleus: Eosin OD min
Smoothed: 50 µm: Nucleus: Eosin OD range
Smoothed: 50 µm: Cell: Area
Smoothed: 50 µm: Cell: Perimeter
Smoothed: 50 µm: Cell: Circularity
Smoothed: 50 µm: Cell: Max caliper
Smoothed: 50 µm: Cell: Min caliper
Smoothed: 50 µm: Cell: Eccentricity
Smoothed: 50 µm: Cell: Hematoxylin OD mean
Smoothed: 50 µm: Cell: Hematoxylin OD std dev
Smoothed: 50 µm: Cell: Hematoxylin OD max
Smoothed: 50 µm: Cell: Hematoxylin OD min
Smoothed: 50 µm: Cell: Eosin OD mean
Smoothed: 50 µm: Cell: Eosin OD std dev
Smoothed: 50 µm: Cell: Eosin OD max
Smoothed: 50 µm: Cell: Eosin OD min
Smoothed: 50 µm: Cytoplasm: Hematoxylin OD mean
Smoothed: 50 µm: Cytoplasm: Hematoxylin OD std dev
Smoothed: 50 µm: Cytoplasm: Hematoxylin OD max
Smoothed: 50 µm: Cytoplasm: Hematoxylin OD min
Smoothed: 50 µm: Cytoplasm: Eosin OD mean
Smoothed: 50 µm: Cytoplasm: Eosin OD std dev
Smoothed: 50 µm: Cytoplasm: Eosin OD max
Smoothed: 50 µm: Cytoplasm: Eosin OD min
Smoothed: 50 µm: Nucleus/Cell area ratio
Smoothed: 50 µm: Nearby detection counts

Supplementary Figure S1

(a) In each whole slide image, the entire tumor region of one whole image selected by the pathologists for each patient was annotated and analyzed. The details of tumor region areas analysed in the (b) RHWU cohort and (c) TCGA cohort are shown.

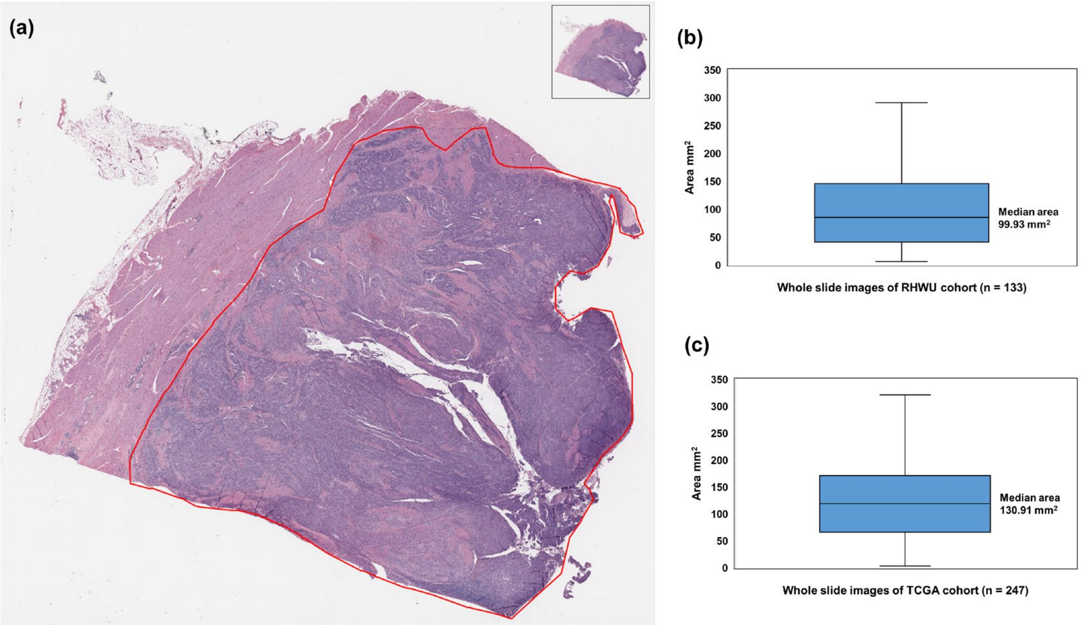

Supplement: Supplementary file 1 [file jcm-11-07081-s001.zip › jcm-2015631-supplementary.pdf]
